# Assessment of diastolic dysfunction in the transplanted heart with analysis of pixel-based deformation fields on CMR

**DOI:** 10.1186/1532-429X-15-S1-E47

**Published:** 2013-01-30

**Authors:** Parag Amin, Xiaoming Bi, Christoph Guetter, Marie-Pierre Jolly, Marius Cordts, Robert A Gordon, Benjamin H Freed, James Carr, Jeremy Collins

**Affiliations:** 1 Radiology, Northwestern Memorial Hospital and Northwestern University Feinberg School of Medicine, Chicago, IL, USA; 2 Cardiovascular MR R&D, Siemens Healthcare, Chicago, IL, USA; 3 Siemens Corp., Corporate Technology, Princeton, NJ, USA; 4 Cardiology, Northwestern Memorial Hospital and Northwestern University Feinberg School of Medicine, Chicago, IL, USA

## Background

Diastolic dysfunction is often used as a marker for allograft rejection and may even precede systolic abnormalities. However, diastolic indices provided by echocardiography can be of inconsistent quality with low sensitivities. Cardiac magnetic resonance (CMR) with high temporal resolution cine imaging offers an alternate noninvasive method for evaluating ventricular function. The purpose of this study is to describe CMR LV diastology indices in a cohort of heart transplant patients, comparing CMR results to echocardiography.

## Methods

A retrospective search was performed for cardiac transplant patients with echocardiograms and CMR examinations with high temporal resolution steady state free precession (SSFP) cine images in long axis orientation (average TR: 11.86 ms) obtained within 1 week of each other. The study cohort comprised 18 patients (14 males, average age 49.0 yrs; 4 females, average age 55.5 yrs). SSFP cine images were analyzed using prototype software evaluating deformation fields to automatically identify and track the mitral base plane at lateral and septal insertions, extracting lateral e' values (Siemens Corp., Corporate Technology; Princeton, New Jersey). Lateral e' values were obtained for each patient from available echo data as well. Additionally, in 13 patients, 3-chamber high temporal resolution SSFP cine images were adequate in orientation for calculation of time elapsed between aortic valve closure and mitral valve opening (IVRT). Bland-Altman analysis was performed. Lateral e' values were reviewed between methods and across estimates of LV filling pressure with E/lateral e' ratios obtained from echo to determine a threshold CMR e' value suggestive of elevated LV filling pressures.

## Results

**Figure 1:**
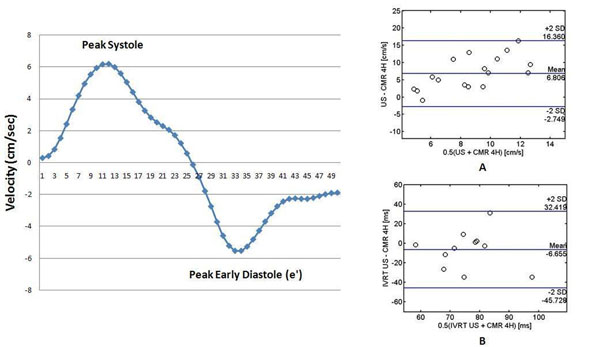
a. Mitral annular velocities measured at lateral LV wall over time through systole and diastole of a representative patient. Figure 1 b. Bland-Altman agreement plots of lateral e' values (A) and IVRT values (B) obtained from echocardiography and CMR on cardiac transplant patients.

Representative lateral mitral annular velocities are shown in Figure 1a. Bland-Altman analysis demonstrates that CMR consistently underestimates lateral e' velocities compared with echocardiography with a trend to increasing differences between methods with increasing e' values (Figure 1b). Bland-Altman agreement plot of isovolumetric relaxation time (IVRT) reveals a small bias of -6.7 ms (Figure 1b), suggesting CMR may underestimate IVRT as well. Comparison of CMR lateral annular e' values with echocardiographic E:e' ratios suggests that a threshold CMR lateral annular e '< 5 msec may correlate to an echocardiographic E/lateral e' > 15 (Table 1).

**Table 1:** Average lateral e' values by imaging method across graded estimates of LV filling pressures with E/e' values obtained from echo on patients with cardiac transplantation.

	Average lateral e' (cm/sec)	
Mitral inflow (E) to annular (e') velocity ratio (from echo)	Echo	CMR

E/e' <8 (n=9)	15.4	5.2
E/e' 8-15 (n=8)	8.7	5.1
E/e' > 15 (n=1)	6	3.7

## Conclusions

High temporal resolution SSFP cine imaging enabled semiautomated quantification of LV diastolic function parameters in heart transplant patients. While CMR consistently underestimated lateral e' values in our cohort, high temporal resolution SSFP cine imaging provided acceptable estimates of IVRT. Additionally, there may be a threshold lateral e' value at CMR (5 ms in our small cohort) that is suggestive of elevated LV filling pressures. Our conclusions are limited due to the small number of patients in this retrospective study; work is ongoing to confirm our findings in a larger cohort.

## Funding

Dr. Collins has funding from RSNA Research & Education Foundation.

